# The Origin of Medical Science

**Published:** 1885-04

**Authors:** 


### THE ORIGIN OF MEDICAL SCIENCE.

We wish that every one could read the address of Prof. Whittaker, published in that excellent journal, *The Cincinnati Lancet and Clinic*, for March 14th, that they might learn from what a maze of superstition and ignorance medical science has been evolved. Its origin seems to have been with the priesthood of antiquity, who pretended to medical lore that they might the more easily hold the people in religious bondage. To this day the clergy love to dabble in empirical medicine, to assert the supremacy of theology over practical science, of metaphysical speculation over physical demonstration. Many a poor sufferer has owed his death to the meddlesome interference of a medically ignorant priest. The clergy may be excellent advisers concerning spiritual affairs, but they usually make sad blunders when they meddle with physics.

